# Les différents modèles de revues scientifiques

**DOI:** 10.48327/mtsi.v3i4.2023.454

**Published:** 2023-12-08

**Authors:** Jean-Philippe CHIPPAUX

**Affiliations:** SFMTSI Société francophone de médecine tropicale et santé internationale (ancienne SPE), Hôpital Pitié-Salpêtrière, Pavillon Laveran, 47-83 boulevard de l'Hôpital, 75651 Paris Cedex 13, France

**Keywords:** Revue scientifique, Société savante, Académie, Recherche scientifique, Frais de publication, Science ouverte, Publication en libre accès, Libre accès aux données, Intégrité scientifique, Évaluation par les pairs, Rédacteur scientifique, Bibliothèque universitaire, Archive ouverte, Internet, Scientific journal, Learned society, Academy, Scientific research, APC, Open access, Open science, Data access, Scientific integrity, Peer review, Scientific editor, Publisher, University library, Open archives, Internet

## Abstract

**Introduction:**

Les revues scientifiques constituent le principal outil de transmission des idées et données scientifiques, dont elles assurent l'enregistrement, la validation, la diffusion et l'archivage. Elles sont aussi un objet économique qui représente un chiffre d'affaires mondial annuel de 25 milliards de dollars expliquant leur positionnement particulier dans la production scientifique.

**Concepts sous-jacents aux modèles des revues scientifiques:**

L'accès aux publications scientifiques (ou science ouverte) s'est particulièrement développé avec la généralisation d'internet et la reconnaissance de la science comme bien commun.

L'intégrité scientifique est contrôlée par les institutions et vérifiée lors du processus d’évaluation.

**Description des modèles de revues scientifiques:**

Deux principaux modèles s'opposent. Le modèle historique qui remonte au XVII^e^ siècle est financé par les lecteurs. Le modèle auteur-payeur, issu du développement d'internet et du concept d'accès ouvert à la littérature scientifique, repose sur le paiement de frais de publication permettant le libre accès du lecteur à la publication et l'autorisation d'utiliser son contenu sous réserve d'en citer la source. Ce dernier modèle se décline en plusieurs versions intermédiaires. Les revues prédatrices, une dérive délétère en expansion depuis 2010, sont caractérisées par lopacité de leur fonctionnement dont l'absence d’évaluation des manuscrits.

L'objectif de chaque revue est alors d'assurer sa propre visibilité qui résulte du nombre de citations davantage que de la qualité des articles.

**Évaluation par les pairs:**

Il s'agit d'un concept ancien dont la mise en œuvre reste complexe, notamment en raison de la difficulté de sélection des évaluateurs.

**Coût et financement des revues scientifiques:**

Les charges varient selon les conditions et les lieux de production. Auteurs et évaluateurs sont bénévoles. En revanche, la gestion, l’édition, la diffusion et l'archivage des manuscrits entraînent des charges financières, généralement inférieures lorsqu'ils sont réalisés dans les pays à ressources faibles ou moyennes. Conclusion. L’édition scientifique cherche un modèle vertueux, financièrement indépendant, qui respecte la science ouverte, le libre accès aux données et l'intégrité scientifique. Son meilleur représentant est le modèle « libre accès diamant ».

## Abstract (en)

**Introduction:**

Scientific journals are the main source of scientific data, ensuring their registration, validation, distribution and archiving. With over 2.6 million scientific articles published each year, the turnover of scientific journals exceeds $25 billion annually. Five publishers share nearly half of this lucrative market. Scientists are the key players in the process, but other stakeholders have gradually been introduced, building various business models whose similarities and differences are described here.

**Concepts underlying scientific publication:**

Open access to scientific papers dates back to the scientific and technical revolution of the 17^th^ century. However, its evolution has been considerably boosted by the development of the Internet and the recognition of science as “commons”.

Scientific integrity is under the control of research institutions to ensure the prevention of fraud and misconduct in the course of scientific production. Usually, the scientific integrity is questioned during the manuscript reviewing process which may result in identification of flaws.

**Models of scientific publications:**

In the historical model, readers pay for access to the document. Authors are not remunerated and renounce copyright on their articles to the publisher. The limits of the historical model became clear in the 90s, facing the cost of publishing, reduction in the number of subscribers, development of the Internet and willingness to improve manuscript evaluation.

With the development of the Internet and the paradigm of open access, publishers proposed a new model in the 2000s, replacing the cost of access to articles for the reader with the payment of Article Processing Charges (APCs) paid by the author or its institution (“author pays” model). In this model, the content of the article can be freely reproduced and used, provided that the original author is credited. In addition to the evaluation of the manuscript which remains a critical factor, the cost of publication appears inequitable. However, all or part of the APCs may be waived, particularly for authors from low- and middle-income countries.

For the past 15 years or so, publishers, learned societies and academic or research institutions (including libraries) have been seeking to publish reliable, open access manuscripts that respect scientific integrity while being affordable for the author.

Predatory journals emerged in the late 2000s, taking advantage of the success of the authorpays model to capture APCs. Lacking a proper evaluation process resulting in poor-quality publications, these journals are rejected by most scientific institutions. On the other hand, they are particularly attractive in low- and middle-income countries because of their aggressive commercial practices (insistent invitation to submit a manuscript, low rejection rate, rapid publication, reduced APC, etc.).

The purpose of each journal is to secure its economic model. This goes through ensuring its visibility, which is determined by the number of citations (online and social media citations) rather than the quality of the articles published.

**Peer review:**

This very old concept has not been widely used until the 20^th^ century. In the historical model, manuscript evaluation is generally carried out by members of the learned society that publishes the journal. Evaluation can be either unblinded, single-blinded (referee is anonymous) or double-blinded (author and referee are anonymous). Several studies have shown that blind procedures do not alter the quality of the evaluation. Since the early 90s, post-publication evaluation has emerged, of which there are several variants. The aim is to shorten times to publication and open up the evaluation process more widely in order to limit the bias. Apart from the fact that this system does not guarantee a better evaluation of the manuscript, its main disadvantage is that the article is accessible without validation of the data collection and analysis throughout the entire process, which can be lengthy.

**Cost and funding of scientific journals:**

The cost of an article depends on charges that vary according to the conditions and places of production. Reviewers are volunteers. On the other hand, manuscript management, editing and distribution are carried out by professionals, which entail financial charges. Some of these costs are lower in low- and medium-resource countries, where economies of scale and higher benefits are possible.

**Conclusion:**

The limits of the historical model have led to the development of several business models of scientific journals, that are in constant evolution, especially the author pays model which promises open access to publications but impacts scientific production. However, the evaluation of scientific production is heterogeneous due to a limited pool of reviewers inadequately selected. Scientific publishing is looking for solutions to find a virtuous model that respects open science, open access to data and scientific integrity. The “Diamond open access” model, free of charge for both readers and authors with the guarantee of an irrevocable license to reproduce the content of the article provided that the original source is cited, perfectly fits.

## Introduction

Indispensable aux échanges d'idées et à leur évolution – donc au développement de la science et de ses produits – la publication scientifique est devenue un marché considérable. Avec plus de 2,6 millions d'articles et 1,4 million d'ouvrages scientifiques publiés en 2019, le chiffre d'affaires dépasse annuellement 25 milliards de dollars avec des bénéfices qui atteignent 40% chez certains éditeurs [9, 13, 22, 37]. Ainsi, les revues scientifiques présentent une double face: outil de transmission du savoir pour les scientifiques qui veulent actualiser leurs connaissances ou partager leurs résultats, d'un côté et objet économique, de l'autre. Cinq éditeurs détiennent près de la moitié de ce marché lucratif et publient environ 6 000 titres parmi lesquels une majorité de revues à facteur d'impact élevé, laissant aux 10 000 autres éditeurs – avec plus de 60 000 revues – ce que l'on appelle la « longue traîne » [9, 22].

**Figure 1:**
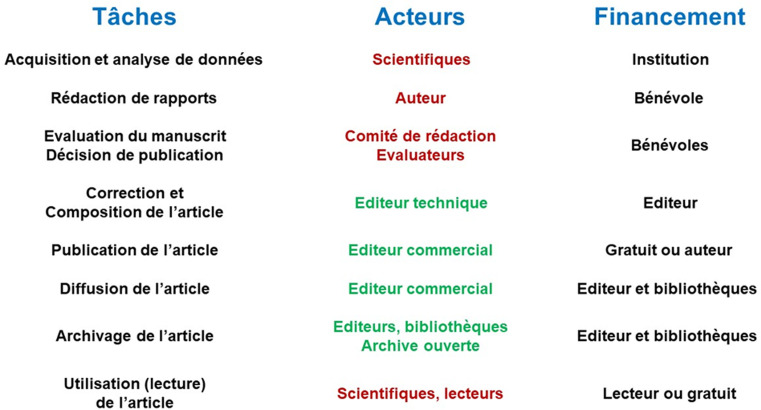
Description du processus de publication; en rouge = communauté d'acteurs scientifiques, en vert = communauté d'acteurs professionnels [d'après 8] Description of the publication process; in red = scientific community, in green = professional community [after 8]

La production scientifique est synthétisée dans un document consignant les résultats et leur interprétation pour en déduire des explications et leçons pertinentes, notamment sous la forme de recommandations de mise en œuvre d'intervention ou de fabrication d’équipements et de produits. Le format du document – qui ne devrait pas influer sur la rigueur de la démarche scientifique – est généralement conditionné par les objectifs visés et un cahier des charges spécifique: littérature grise, brevet, mémoire, thèse, livre, chapitre de livre, film, communication à un congrès (orale ou affichée), article original publié dans une revue (imprimée ou en ligne). La publication de ce rapport résulte d'un enchaînement de tâches faisant appel à des personnels appartenant à des communautés d'intérêt différentes (Fig. 1) [8]. L'article a longtemps été privilégié en tant que standard de la production scientifique, à la fois comme référence et indicateur d'activité professionnelle (Publier ou périr^1^). À cet égard, on peut considérer que le scientifique, ingénieur ou chercheur, fabrique un produit destiné à devenir un bien de consommation qui sera jugé et utilisé en fonction de sa pertinence et de sa rentabilité. Toutefois, contrairement à ce que l'on pourrait croire, il n'y a pas de véritable concurrence entre les éditeurs en raison du manque de substituabilité du produit: une revue est composée d'articles originaux dont aucun ne peut être remplacé par un autre [16, 29]. Cette absence de concurrence déséquilibre les rapports de force entre les acteurs.

Depuis le xvii^e^ siècle, initialement gérées par les sociétés savantes, les revues scientifiques constituent le principal outil de transmission des résultats scientifiques, dont elles assurent l'enregistrement, la validation, la diffusion et l'archivage. La publication est devenue un indicateur incontournable du fonctionnement de la science et de la carrière du chercheur [7, 29]. Si le scientifique est la cheville ouvrière du processus, d'autres acteurs se sont imposés progressivement, notamment les rapporteurs, évaluateurs, comités de rédaction, imprimeurs, entreprises d’édition, universités, bibliothèques, agences d’évaluation, publicitaires, etc. [16, 26]. Ils ont façonné des modèles plus ou moins appropriés, mais rentables (au moins pour certains), laissant parfois la science sur le bord du chemin.

Plusieurs modèles de revues scientifiques coexistent avec de nombreux intermédiaires entre le modèle historique, prépondérant jusqu’à la fin des années 90, dans lequel le lecteur est le payeur, et le modèle qui se développe depuis une vingtaine d'années où l'auteur, une fois l'article accepté, paye pour être publié afin que l'article soit en accès libre. Nous en discuterons les points communs et les divergences.

## Les paradigmes de science ouverte, de libre accès aux données et d'intégrité scientifique

Le souhait de rendre le produit de la recherche accessible à tous remonte à la révolution scientifique et technique du xvii^e^ siècle [14]. Ce vœu a semblé devenir réalisable avec l'avènement simultané, d'une part d'internet et, d'autre part de la reconnaissance de la science comme « bien commun » [14, 24].

Une distinction doit être faite entre les notions d'accès ouvert et de libre accès^2^. Le premier fait référence à un accès gratuit, indépendamment du droit de réutilisation. Le second accorde, en plus de l'accès ouvert, un droit d'utilisation du contenu grâce à une licence spécifique exigeant au minimum la mention de l'origine du document. En pratique, ces deux notions sont souvent synonymes, d'autant que les droits d'utilisation sont souvent limités, notamment pour ce qui concerne les données brutes [33]. Depuis quelques années, l'acronyme FAIR pour *findable, accessible, interoperable and reusable* (= trouvable, accessible, interopérable et réutilisable) reflète ce que l'on entend par libre accès.

Le concept de science ouverte s'est structuré à partir des années 2000. S'adossant, notamment, aux revues scientifiques en accès ouvert, il a intégré les formes numériques de collaboration ouverte et d'aménagement du droit d'auteur^3^ pour élargir l'exploitation des résultats et l'utilisation des protocoles scientifiques [14]. Depuis peu, des articles spécifiques décrivent le protocole de recherche d'une étude en projet, permettant leur validation indépendante préalablement à l’étude elle-même, et l'allègement ultérieur de la présentation des résultats [25].

Le libre accès aux données est rarement réalisé de façon satisfaisante. D'une part, les données sont soumises à des enjeux financiers complexes. D'autre part, les auteurs restent méfiants quant au partage de leurs données. Leur présentation dans les articles est bien souvent tronquée et inexploitable, volontairement ou non. Il existe, par ailleurs, des plateformes dédiées où les données sont conservées et organisées pour stimuler leur utilisation [33]. Ce processus, pour séduisant qu'il soit, ne garantit pas la qualité du recueil de données et leur représentativité.

L'intégrité scientifique, qui est indissociable de la déontologie de la recherche, constitue depuis peu une obligation légale dans de nombreux pays, y compris en France. Son contrôle est exercé par les institutions concernées afin d'assurer la prévention de la fraude et de toute inconduite au cours de la production scientifique [2, 12]. Cependant, c'est généralement lors du processus d’évaluation d'un manuscrit que l'intégrité scientifique est interrogée et que sont identifiés, si c'est le cas, la fabrication ou la falsification des données, le plagiat, l'embellissement, la sélection ou la non-reproductibilité des résultats, l'utilisation incorrecte des tests statistiques, l’émiettement des publications, les conflits d'intérêts et les signatures abusives, manquantes ou dans un ordre inadéquat. L'impartialité et l’équité de l’évaluation scientifique sont garanties par les éditeurs scientifiques, mais les éditeurs technico-commerciaux ne sont pas dispensés d'une vigilance renforcée à cet égard [2].

Historiquement réalisé par les revues ellesmêmes, l'archivage s'est développé avec la science ouverte grâce, notamment, à internet qui permet la mise en ligne rapide des articles, mais aussi le stockage et le partage des données, ou le dépôt des publications dans un site d'archivage dédié. Le fonctionnement des revues scientifiques s'est simplifié et les procédures des échanges, notamment la publication et la diffusion des articles se sont accélérées [16]. Les archives institutionnelles sont gérées par une personne morale (une institution de recherche ou une université) dont l'objectif principal est la conservation du patrimoine de ses ressortissants, ce qui peut restreindre l'accès à certains documents. Plus récemment se sont développées des archives ouvertes libres, bases de données à la fois trans-institutionnelles et transdisciplinaires, organisées par des entités nationales (H AL en France) ou internationales (arXiv par Cornell University et PubMed’ par les National Institutes of Health) [38]. Pour l'essentiel, ces archives sont tributaires de leur alimentation, le plus souvent laissée à l'initiative des auteurs.

## Modèle historique

**Figure 2:**
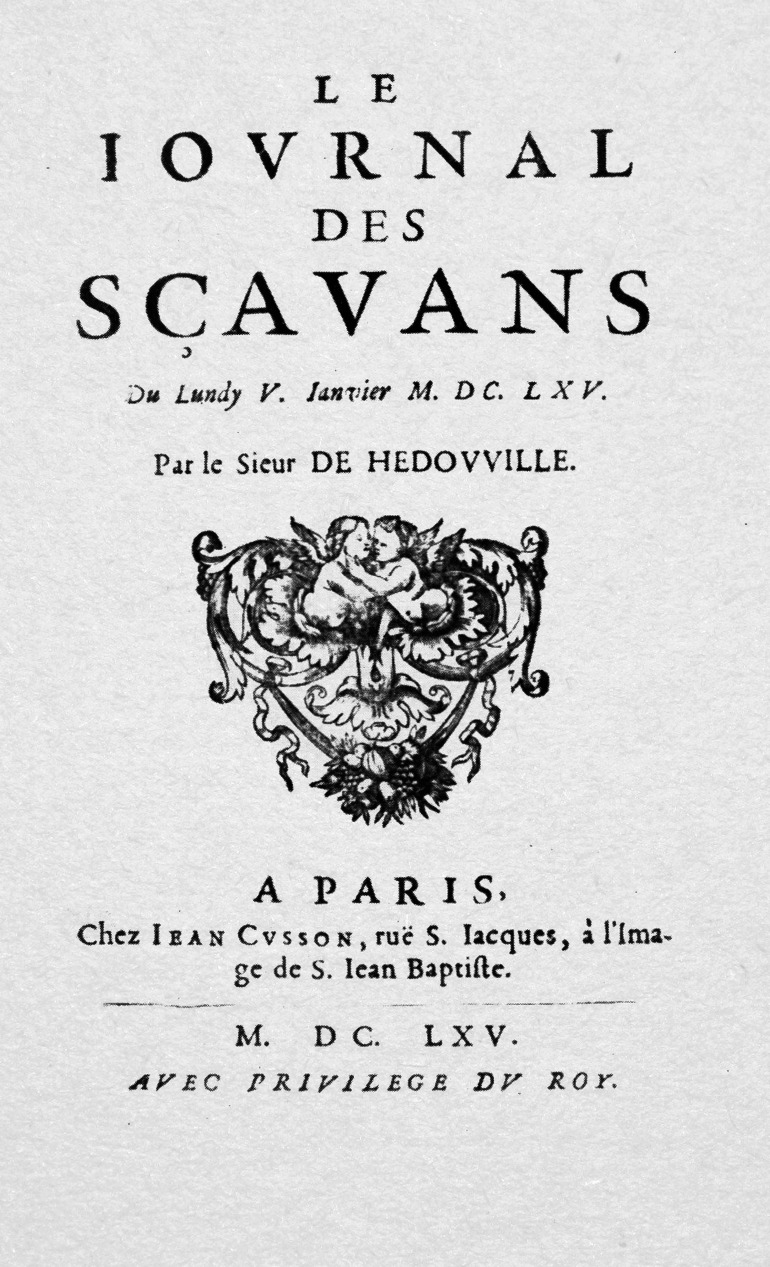
Couverture du premier numéro du Journal des Sçavans paru le 5 janvier 1665 (Source Wikipédia) Cover of the first issue of the Journal des Sçavans published on January 5, 1665 (Source Wikipédia)

**Figure 3:**
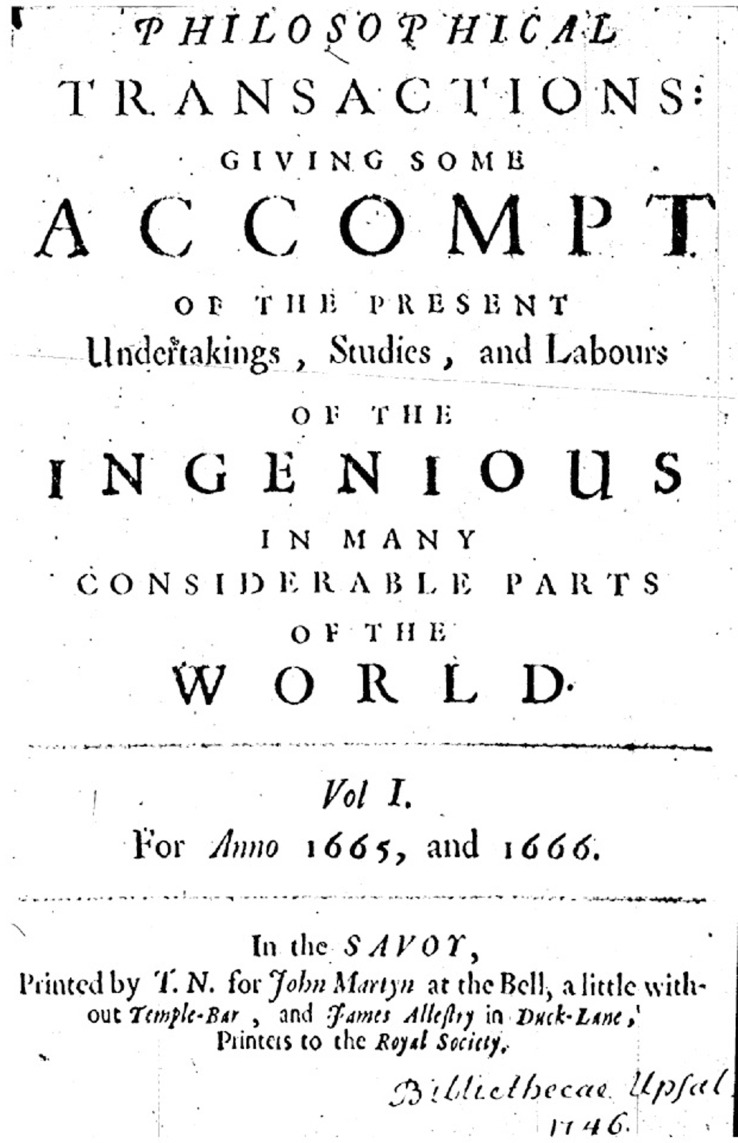
Couverture du premier numéro des Philosophical Transactions of the Royal Society paru le 6 mars 1665 (Source Gallica) Cover of the first issue of the Philosophical Transactions of the Royal Society published on March 6, 1665 (Source Gallica)

Encore appelé modèle « payer pour voir », « abonné payeur » ou « sponsor », ces revues sont majoritaires. Elles représentent encore près des deux tiers de l'ensemble des publications scientifiques périodiques. Apparues simultanément en 1665 à Paris et Londres, deux d'entre elles perdurent encore aujourd'hui [17, 26, 41]. Le *Journal des sçavans* parut pour la première fois le 5 janvier 1665^4^ (Fig. 2), tandis que la première livraison des *Philosophical Transactions of the Royal Society* date du 6 mars 1665 (Fig. 3). Leur nombre a été en constante augmentation jusqu’à la fin du xx^e^ siècle, avant l'arrivée de revues appartenant à d'autres modèles.

La publication est gratuite pour l'auteur qui, dans la plupart des cas, cède ses droits à la société savante [15]. En revanche, les articles sont payants pour le lecteur qui doit, pour y avoir accès, être abonné à la revue ou aller la consulter dans une bibliothèque pour qui le tarif est généralement très supérieur à celui de l'abonnement individuel.

À l'origine, l'ensemble des tâches – en dehors de la composition et de l'impression de la revue qui revenaient à l'imprimeur – était assuré bénévolement par les membres de la société savante. Le rédacteur (en anglais *editor*) scientifique, représenté par le comité éditorial appuyé par un conseil scientifique^5^, évalue le manuscrit et décide de sa publication. Il joue aussi le rôle d’éditeur technicocommercial – appelé *publisher* en anglais [4] – qui supervise la composition, l'impression, la diffusion et l'archivage de la revue, ainsi que les abonnements aux bibliothèques, en faisant éventuellement appel à des salariés ou des prestataires extérieurs. L’évaluation du manuscrit, dont le processus sera décrit plus loin, était l'apanage du rédacteur en chef ou du comité de rédaction qui s'en est d'abord occupé seul puis a fait appel à des évaluateurs^6^. Les limites du modèle historique sont apparues dans les années 90 avec, notamment, le coût croissant de l’édition, la réduction inexorable des abonnés, le photocopillage, le développement d'internet et le souhait d'améliorer l’évaluation des manuscrits [34].

## Modèle auteur-payeur

Face à la croissance exponentielle des publications scientifiques et attirés par la perspective de profits substantiels, les éditeurs privés ont investi les revues scientifiques. S'appuyant sur l'apparition de nouveaux paradigmes notamment ceux de la science ouverte et le « libre accès aux données » décrits plus loin –, ils ont élargi le périmètre du financement de la publication scientifique et introduit le concept d'auteur-payeur^7^. Ce renversement audacieux de situation n'a pas été sans surprendre [15, 16, 28].

Le pas a été sauté symboliquement en janvier 2001 par une pétition en ligne de la *Public Library of Science (PLoS)* réunissant 34 000 signatures de scientifiques de 180 pays [8, 40].

La pétition revendiquait un libre accès aux archives permanentes de la recherche afin que la production scientifique ne soit pas contrôlée par les éditeurs mais appartienne au public via une bibliothèque publique internationale en ligne. Celle-ci, qui concerne les articles publiés dans les revues d'un même éditeur, se distingue de l'archive ouverte qui sera décrite plus loin. Faute d’éditeur technico-commercial, les premiers articles de *PLoS* qui devaient paraître en septembre 2001 ont été mis en ligne le 13 octobre 2003 *(PLoS Biology,* immédiatement suivie par quatre autres revues, dont *PLoS Medicine* le 19 octobre 2004). Le contenu des articles publiés par *PLoS* est régi par les termes de la licence *Creative Commons Attribution* (CC BY) selon laquelle le contenu de chaque article peut être utilisé librement à condition que la source originale et les auteurs soient mentionnés explicitement. Dix ans après sa création, *PLoS Biology* dégageait des bénéfices, confirmant la viabilité économique de ce modèle [1]. De nombreuses autres revues ont suivi ce modèle.

La gestion du manuscrit, sa publication, sa diffusion et son archivage sont assurés par une équipe de professionnels rémunérés, complètement indépendante de l’évaluation et de la décision de publication (ou non) réalisées par des bénévoles, à titre individuel ou au nom d'une société savante. Ce modèle accélère significativement le processus de fabrication et de diffusion des articles, notamment grâce à internet, processus qui était une contrainte importante du modèle historique, un délai raccourci de publication étant un atout essentiel de la communication scientifique. Cependant, il ne parvient pas à réduire le temps d’évaluation du manuscrit, ni à en améliorer le résultat comme nous le verrons.

La mainmise par les professionnels de l’édition sur le fonctionnement des revues présente l'avantage de soulager les scientifiques, généralement peu compétents dans ce domaine, d'améliorer la présentation des articles, ainsi que la gestion administrative et financière de l'ensemble. La rentabilité du modèle résulte de la mutualisation des moyens mis au service d'un nombre croissant de revues, ce qui permet une économie d’échelle significative. L'absence d'influence sur le contenu scientifique est théoriquement préservée par l'indépendance du comité éditorial dont la décision finale est souveraine. Cependant, malgré des règles de fonctionnement très strictes et une rentabilité qui ne fait pas défaut, ce modèle a rapidement montré ses limites. Outre l’évaluation du manuscrit qui reste un élément critique (voir plus loin), le coût de publication, via les frais de publication ou APC^8^ variables en fonction de la notoriété de la revue, apparaît inéquitable [3, 11, 21]. Plus inquiétant, ce modèle ouvre la voie à des dérives perverses comme les revues prédatrices définies ci-dessous [13].

## Modèles alternatifs

Depuis une quinzaine d'années, les éditeurs, les sociétés savantes et les institutions universitaires ou de recherche, sont en quête de compromis favorisant la publication de manuscrits intègres (l'intégrité scientifique est abordée plus loin) et accessibles à tous à moindres frais pour l'auteur. Quelques exemples sont rappelés dans le Tableau I. De nombreuses autres options dérivent du modèle auteur-payeur [4, 13, 32, 38].

**Tableau I:** Les principaux modèles de publications en accès ouvert [4, 13, 26] Main models of open access journals [4, 13, 26]

Modèle	Pour l'auteur	Pour le lecteur	Condition d'utilisation
Noir	Gratuit	Gratuit via un site dédié	Illégale
Vert	Gratuit (rares exceptions)	Gratuit via l'auteur	Archivage par l'auteur
Bronze	Gratuit	Gratuit sur la page de l’éditeur	Pas de licence
Or	Frais de publication	Accès totalement libre	Licence CC BY
Diamant	Gratuit	Accès totalement libre	Licence CC BY

- Le modèle freemium permet au lecteur d'accéder au contenu brut de l'article (au format html, par exemple) mais requiert un paiement pour bénéficier de services complémentaires (accès aux formats pdf ou epub qui permettent l'archivage, aux statistiques d'usage, etc.).
- Le modèle hybride laisse à l'auteur le choix entre deux options: publier gratuitement son article qui sera accessible aux seuls abonnés, ou le mettre en accès ouvert moyennant l'acquittement de frais de publication. De fait, les institutions de recherche, notamment les universités, payent deux fois la revue avec l'abonnement et les frais de publication^9^ [15].
- L'embargo permet d'ouvrir l'accès aux articles à tous après une période de 6 mois à 2 ans pendant laquelle ils sont réservés aux seuls abonnés.
- Le financement participatif propose des projets en ligne (par exemple un numéro spécial composé d'articles sur une thématique commune), incitant la communauté concernée à en financer la publication.
- La dotation réalise un fonds abondé par des contributions destinées à financer les frais de publication. Les dotations peuvent être développées par le biais de campagnes de collecte de fonds ou la vente de produits dérivés (T-shirts, mugs, gadgets divers).
- Dans le modèle par APC institutionnelles, une institution de recherche ou d'enseignement subventionne une revue permettant à ses chercheurs d'y publier gratuitement ou à un tarif minoré.
- Le modèle Diamant est gratuit pour les lecteurs comme pour les auteurs. Les manuscrits sont validés par les pairs et respectent l'intégrité scientifique. Il est intégralement financé par une institution ou une société savante. Ce modèle est parfaitement adapté aux travaux provenant de chercheurs et d'organismes disposant de peu de moyens financiers.

## Dévoiement prévisible: les revues prédatrices

Les revues prédatrices profitent du succès du modèle auteur-payeur pour s'approprier les frais de publication. Il existe de nombreuses définitions qui, toutes, soulignent leur côté mercantile, l'absence de transparence de leur mode de fonctionnement, leur mépris des bonnes pratiques de rédaction et de publication, leur recours à des sollicitations maladroites voire agressives [19, 26]. S'y ajoutent souvent de fausses déclarations sur leur facteur d'impact et d'indexation.

Leur capacité d'adaptation rapide aux particularités du marché rend toute définition et liste superflues voire contre-productives. Grâce à la complaisance de nombreux auteurs à leur égard, elles réduisent la visibilité des revues vertueuses et masquent les dérives, y compris parmi les plus prestigieuses [26, 27]. Cependant, le curseur entre revue prédatrice et revue vertueuse n'est pas fixe. Ainsi, de nombreuses académies et universités suggèrent de remplacer les listes et définitions des revues prédatrices ou vertueuses par un ensemble d'indicateurs permettant de les classer en fonction de leurs caractéristiques et pratiques^10^ [27, 32].

Ces revues sont dénoncées par toutes les instances et institutions scientifiques, particulièrement celles des pays à revenu faible ou intermédiaire^11^ en raison de l'attrait qu'elles représentent pour les scientifiques de ces pays, notamment la simplicité de soumission, la peur d'un refus par une revue qualifiée, leur rapidité de publication^12^ et, lorsqu'ils sont affichés, des frais de publications réduits. En outre, les auteurs ne sont pas informés de l'absence d’évaluation de leur manuscrit et, bien souvent, des frais de publication qui s'appliqueront [6, 23, 25].

## Visibilité des revues

Compte tenu des enjeux économiques et quel que soit le modèle, chaque revue vise à accroître sa visibilité pour attirer auteurs et lecteurs. Celle-ci est imparfaitement traduite par deux outils distincts mais complémentaires: l'indexation et les indices de notoriété [5, 26]. L'indexation correspond au référencement des articles d'une revue dans une base de données indépendante pour les archiver et les rendre accessibles. Elle repose sur une trentaine de critères administratifs ou fonctionnels, notamment le localisateur uniforme de ressource (URL) du site internet, l'adresse physique de l’éditeur, le numéro international normalisé de la publication (ISSN), l'identifiant numérique d'objet (DOI), le mode de financement de la revue, son indexation et ses indicateurs de visibilité ou de notoriété, le format des articles, la composition du comité éditorial et du conseil scientifique, les procédures de soumission et d’évaluation des manuscrits, les recommandations aux auteurs, la politique de rétractation d'articles, la gestion de l'archivage interne des articles, le guide d’éthique, etc. Aucun de ces critères ne concerne directement la qualité scientifique du contenu des articles. Le Directory of Open Access Journals (DOAJ: https://doaj.org) inventorie les revues scientifiques en ligne en se fondant sur 5 critères, ce qui permet d’écarter la plupart des revues prédatrices mais pas toujours les pratiques déviantes [32]:

- les informations de base de la revue concernant les caractéristiques administratives listées ci-dessus;
- la qualité et la transparence du processus éditorial, y compris l'archivage des articles;
- les modalités d'accès aux articles;
- l'autorisation de réutilisation du contenu selon une licence appropriée;
- la politique d'application du droit d'auteur.

Les indices de notoriété, comme le facteur d'impact qui est un argument décisif pour le choix d'une revue par l'auteur et le lecteur, sont fondés sur le nombre de citations des articles de la revue. Les mesures d'impact alternatives^13^ quantifient les récurrences et les interactions que suscite une publication dans les blogs et les réseaux sociaux, le nombre de téléchargements ou la fréquence des signets associés à un URL. Ce marquage varie en fonction du champ disciplinaire, plus important dans les sciences humaines et sociales que dans les sciences biologiques, elles-mêmes davantage que dans les sciences physiques et mathématiques. Aucun ne traduit la qualité scientifique des articles. Ils dépendent de la discipline, du sujet traité et des circonstances qui souvent entraînent des effets de mode et de mimétisme, sur le court terme.

## Évaluation des manuscrits par les pairs

Les revues scientifiques ne se contentent pas de transmettre le savoir. Elles évaluent les résultats de la recherche en mettant en place un processus approprié, même s'il montre des faiblesses. Le principe de l’évaluation par les pairs est très ancien, mais n'a été que peu utilisé jusqu'au xx^e^ siècle [34]. Il s'est construit au sein des sociétés savantes, d'abord de façon informelle et occasionnelle, puis s'est généralisé avant d’être institutionnalisé au cours des années 1960.

Pendant longtemps, les résultats de la recherche étaient présentés avant leur publication aux membres de la société savante lors de réunions périodiques, des questions étaient publiquement posées à l'auteur principal et ces questions et les réponses apportées étaient publiées à la suite des articles, ce qui constituait une première forme d’évaluation, quoique non anonyme. Dans le modèle historique, l’évaluation des manuscrits est réalisée par les membres de la société savante éditrice – dont l'effectif est généralement limité – à défaut par des experts extérieurs identifiés par la rédaction comme spécialistes reconnus du domaine. Ce processus est d'autant plus long que le nombre des évaluateurs est restreint et qu'il peut être difficile de les mobiliser, tous n’étant pas volontaires. Pour raccourcir la durée de l’évaluation, il est nécessaire de recruter un nombre considérable d’évaluateurs représentatifs de tous les aspects de la recherche scientifique dans les disciplines et spécialités couvertes par la revue. C'est l'option retenue par le modèle auteur-payeur. Cependant, ce vivier beaucoup plus important que celui du modèle historique, est hétérogène^14^. Le recrutement des évaluateurs est complexe en raison de la difficulté de mesurer la compétence de chacun en termes d'expertise et de capacité d’évaluation du travail d'un tiers. L'essor de l’évaluation par les pairs a été rendu possible grâce à deux inventions qui ont facilité la reproduction des manuscrits tout en garantissant l'indépendance des avis: la machine à écrire avec la copie carbone en 1890 et la photocopieuse en 1959 [35]. Le développement des techniques, l'extension des disciplines et la spécialisation grandissante ont nécessité d'avoir recours à des experts de plus en plus qualifiés.

Jusqu'en 1990, l’évaluation en simple aveugle, dans laquelle l’évaluateur est anonyme mais l'auteur identifié, a dominé. Il prévaut encore dans le modèle historique et reste utilisé dans la majorité des revues appartenant aux modèles prônant la science ouverte. Pour beaucoup, il favorise l'objectivité et la franchise. Cependant, outre les préjugés et la subjectivité consciente ou non de l’évaluateur lors de l'examen du manuscrit, il existe un risque non négligeable de retard de publication – parfois volontaire –, voire d'utilisation frauduleuse des résultats puisque les deux protagonistes appartiennent à la même discipline et sont susceptibles de travailler sur le même sujet [28, 31, 34]. De plus, l'anonymat de l'examinateur peut l'inciter à être exagérément sévère. À l'opposé, l'absence d'anonymat se traduit généralement par un plus grand laxisme pour éviter d'inutiles contestations, autant de la part des auteurs que des collègues [34]. Il peut rebuter un évaluateur inexpérimenté et l'influencer si l'auteur est connu [31]. L’évaluation en double aveugle, où l'anonymat s'applique à la fois aux auteurs et aux examinateurs, est approuvée par la majorité des chercheurs. Cependant, d'une part il n'empêche pas les commentaires hostiles de l'examinateur et, d'autre part dans des domaines où le nombre de spécialistes est très restreint, l'anonymat est compromis par des opinions revendiquées, un style d’écriture particulier ou la fréquence des autocitations [31].

Plusieurs études ont montré que l'anonymat n'altère pas la qualité de l’évaluation [18, 39]. Toutefois, l’évaluation en double aveugle semble être associée à un taux d'acceptation des manuscrits inférieur de près de 20% à celui de l’évaluation en simple aveugle [36]. Avec l'Open peer review, dont il existe de nombreuses modalités, l'identité des auteurs et des évaluateurs est transparente dès le début du processus. Généralement, les rapports d’évaluation et les différentes révisions du manuscrit sont accessibles sur le site ou la plateforme de publication. Cette forme d’évaluation ouverte est proposée par un nombre croissant d’éditeurs scientifiques comme Bio-Med Central ou PLoS.

Depuis le début des années 1990, l’évaluation postérieure à la publication dont il existe plusieurs variantes, est apparue [25, 26]. L'objectif est de réduire les délais de publication et d'ouvrir plus largement l’évaluation afin d'en limiter les biais. En pratique, l'article – ici appelé prépublication ou preprint – est déposé sur une plateforme après un examen éditorial sommaire ne portant que sur la forme du document [20]. Selon les plateformes, des évaluateurs volontaires ou désignés sont invités à critiquer le manuscrit et faire des recommandations pour l'accepter, l'améliorer ou le rejeter. L'article s'accompagne de l'historique de son évaluation. Sur certaines plateformes, l’évaluation est ouverte à tous les lecteurs qui peuvent intervenir dans un blog. Ce système favorise le dialogue et élargit le nombre d’évaluateurs, donc la diversité des commentaires, permettant l’évolution du contenu de l'article [25]. Cependant, il y a un risque important de perte du contrôle éditorial et de débats prolongés, souvent stériles [31]. Sans garantir une meilleure évaluation du manuscrit, le principal défaut est que l'article est accessible pendant toute la durée de l’évaluation. Les résultats sont donc utilisables avant toute validation, parfois sans que les utilisateurs ne le prennent en compte^15^. Il faut souligner que le délai de publication peut être plus long que la durée d'une évaluation antérieure à la publication. En outre, de nombreuses revues scientifiques refusent les articles diffusés préalablement en preprint, dont elles considèrent qu'ils ont perdu leur originalité, ce qui peut encore retarder la publication de l'article validé le temps d'identifier une revue qui l'accepte.

## Coût et financement des revues scientifiques

Le coût d'un article repose sur des charges variables en fonction des conditions et lieux de production. Bien qu'essentiels, deux postes sont généralement exclus du budget récurrent: la contribution de l'auteur et celle des évaluateurs^16^. Le travail du premier – réalisation de la recherche, analyse des données, rédaction du rapport – est financé par des sources publiques ou privées mais, en principe, toujours indépendantes des éditeurs technico-commerciaux qui exploitent le produit sans y contribuer financièrement. Les seconds – évaluateurs et comité de rédaction – sont bénévoles, ce qui ne rend pas leur participation gratuite pour autant. En revanche, la correction formelle du manuscrit, sa composition pour l’éditer au format final (html, pdf, ePub ou autre) quel que soit son type de diffusion, son impression s'il s'agit d'une édition papier, et son archivage sont facturés (Fig. 1). Ces tâches comprennent les frais de personnel, logiciels, équipements, consommables comme le papier, et diverses charges (locaux, énergie, etc.). Certaines d'entre elles, notamment le personnel, se révèlent moins élevées dans les pays à ressource faible ou modérée, favorisant des économies d’échelle et de meilleurs profits. Il n'est donc pas surprenant que les revues prédatrices y prolifèrent.

Ce marché est structuré autour de l’éditeur technico-commercial et des bibliothèques qui négocient – dans un marchandage inégal le prix des abonnements [16]. Les premiers vendent la réputation de la revue aux seconds qui proposent gratuitement les articles aux lecteurs. Cependant, les maisons d’édition restent maîtresses du jeu puisqu'elles détiennent un portefeuille de revues dont elles maîtrisent la diffusion. Outre le prix de l’édition qui augmente plus rapidement que l'inflation et une baisse constante du budget des bibliothèques, les éditeurs usent de stratégies abusives. La principale est le regroupement des abonnements dans des bouquets (*big deal* ou *bundle*) dont le prix global est réduit par rapport au coût individuel de chaque revue, ce qui permet à la bibliothèque de recevoir les revues demandées à condition de s'abonner aussi à de nombreuses revues numériques dont elle n'a pas besoin [4, 13, 28, 29].

## Conclusion

L'essoufflement du modèle historique qui dépend entièrement des abonnements, a permis le développement d'autres modèles de revues scientifiques comme celui de l'auteur-payeur qui promet un accès ouvert aux publications mais pèse sur la production scientifique. Quels que soient la notoriété de la revue scientifique et son mode de diffusion, il existe de nombreux modèles en perpétuelle évolution [9, 16, 25]. Leur visibilité dépend de la fréquence de citation de leurs articles et non de leur qualité scientifique.

Jamais rémunéré pour la rédaction d'un manuscrit original, l'auteur doit faire face à une alternative qui l'oblige, soit à acheter l'article scientifique dont il a besoin pour travailler, soit à payer pour publier ses résultats. Ainsi la logique de l’édition scientifique internationale correspond-elle à l'archétype du sous-développement caractérisé par la production de matière première à prix bas et à l'importation de produit manufacturé à coût élevé [15]. Dans ce contexte, il paraît logique que le financement des revues scientifiques, adossé à celui de l'accès ouvert, fasse largement appel aux subventions publiques et privées selon différentes voies. Cette politique est légitime sous réserve de maintenir l'indépendance des éditeurs scientifiques et technico-commerciaux vis-à-vis du financeur.

La question de l’évaluation des manuscrits subsiste. Sa qualité est hétérogène, y compris au sein de la même revue, en raison d'un vivier d’évaluateurs restreint, en particulier dans le modèle historique, et d'une sélection inadéquate des évaluateurs dans tous les modèles, basée sur leur notoriété, le nombre de leurs publications et leur disponibilité – voire leur vanité que les éditeurs commerciaux savent flatter parfois sans subtilité – plutôt que sur leur capacité à juger un travail ou un manuscrit. Face à une offre en constante augmentation, la publication scientifique cherche un modèle qui respecte les principes de la science ouverte, de l'accès libre aux données et de l'intégrité scientifique pour parvenir à un modèle vertueux. Le modèle de publication en libre accès diamant, c'est-à-dire gratuit pour les auteurs et les lecteurs (licence CC BY), répond parfaitement à cet objectif sous réserve qu'il bénéficie d'un financement pérenne dont il doit rester indépendant.

Cependant, la publication et la diffusion de l'article – même réussies – ne résolvent pas la question cruciale de son impact, à supposer qu'il soit lu…

## Liens d'intérêts

L'auteur ne déclare aucun conflit d'intérêts.
